# Ethics in medical curriculum; Ethics by the teachers for students and society

**DOI:** 10.4103/0970-1591.56192

**Published:** 2009

**Authors:** Karuna Rameshkumar

**Affiliations:** Department of Clinical Pathology, John's Medical College, Bangalore 566 034, India

**Keywords:** Medical ethics

## Abstract

There are many ethical issues involved in the practice of modern medicine. It can be a simple one-on-one issue with complex ramifications. The training of medical ethics should be a continuous process. The ideal time to introduce ethics is a subject of many debates. Though it has to be introduced during the undergraduate curriculum, it requires reinforcing during specialty training also. The teaching of medical ethics can utilize various methodologies. There should be a proper evaluation of the ethical aspects learned.

In Europe and the U.S.A., many institutions offer national integrated programs in medical ethics education. The main motivation for such programs is to focus and train the new academicians, bureaucrats, and legal experts who are needed to cater to the growing demand for technical aspects of knowledge in bioethics.[1] This includes lawyers, specialists in patenting issues, public relations, and administration. Hence, most of the programs are need-based structured programs. To a certain extent, the popularity of ethics is also due to the outlet provided by debates and deliberations on ethics without having to engage in legal tussles. But it is not enough to have deliberations without a well-defined outcome. The dissemination of information and education has to be undertaken in a structured manner in order to ensure messages are not distorted and that objectives are truly attained. In India, though the perceived need is always there, very few institutions offer such structured programs.

The training of medical ethics is a continuous process and it does not always happen. Ethical issues have become complex, profound, and require careful investigation to find the right answers. The time to introduce the content of a medical ethics curriculum has been the subject of discussion in many studies.[2–4] The history of medical ethics in India began at St. John's Medical College, which was one of the first institutions at St. John's National Academy of Health Sciences dating back to 1963. As recently as 1998, St. John's Medical College was the only Medical College in India teaching medical ethics as a regular part of its undergraduate curriculum. Some of the topics are addressed by the Department of Forensic Medicine. Interns are required to attend monthly clinical ethics sessions in which cases involving ethical issues are presented and discussed by faculty and members of the department of medical ethics. The Rajiv Gandhi University of Health Sciences (RGUHS), to which the college is affiliated, has recently incorporated medical ethics into its syllabus, using the St. John's template and requiring 40 hours over the period of the MBBS program. Phase I of the 6 hours in the preclinical period has to be covered by the departments of anatomy, physiology, and biochemistry and Phase II of the 6 hours in the paraclinical period are covered by pharmacology, pathology, and microbiology departments. In Phase III, 28 hours have to be covered by the opthalmology, ENT, medicine, surgery, OBG, and other clinical departments. These will be achieved through classroom teaching, bedside teaching, demonstrations by examples and interactions with patients, patient relatives, colleagues and the public.

The undergraduate medical curriculum of Toronto University covers ethics topics in 50 hours.[5] Since 2004, the Indian Council of Medical Research (ICMR) has been conducting awareness and training workshops for students as well as faculty throughout the country. This has created a tremendous interest in medical ethics among the students and the medical fraternity. The Medical Council of India (MCI) has published “Regulations relating to the professional conduct, etiquette, and ethics for registered medical practitioners”.[6] This replaces an earlier version published as far back as 1970. This new code of ethics has rules for medical practice organized into eight chapters. There has been a general apprehension that the code of ethics modified by MCI in India is admirable in paper but is sorely in need of strict implementation.[7] Now, many universities and medical colleges are taking efforts to introduce it into the curriculum though the efforts do not match the need and the perceived gaps in medical education.

In his observations, Singer had suggested that the best time to teach medical ethics may be during postgraduate education or continuing professional development.[8] The Royal College of Physicians and Surgeons of Canada requires medical ethics to be taught as a condition of accreditation of a postgraduate program and has developed model curriculums in various specialties.[9] A study of 16 teaching hospitals that provide a general internal medicine residency program in Japan showed that 75% of participant postgraduates wanted to have a more comprehensive education in medical ethics.[10] The results suggested that many residents desired more comprehensive and interdisciplinary education in medical ethics and educators in Japan should aim to develop education program to meet these needs. If ethics teaching is reinforced at the postgraduate level, instead of being introduced only at that level, it may be expected that physicians, having completed their training, will be more aware of and sensitive to the ethical dimensions of practice of medicine.

The structure of ethics education has to be closely monitored and the curriculum goals have to be well defined. The ultimate objectives will be to make the students recognize the humanistic and ethical components of health care and to translate and integrate ethical principles into clinical practice.[11] Standard ethics that help to guide behavior, actions, and choices can be viewed from two angles – normative and prescriptive. Normative refers to well-based standards of right and wrong that prescribe what humans ought to do in terms of rights, obligations, benefits to society, fairness, and specific virtues. Prescriptive refers to the study and development of ethical standards, as well as community ethics. Approaches based on moral reasoning have an important limitation: they are only one piece of the puzzle. To address effectively the disclosure of bad news, informed consent, confidentiality, dishonesty, research ethics, end-of-life care, resource allocation and the like, the doctor must recognize situations as an ethical dilemma, possess the relevant knowledge of norms, laws and policies, analyze how this knowledge applies to the situation at hand, and demonstrate the skills needed to communicate and negotiate this situation in practice. When medical ethics is taught by clinicians, it has an advantage because they not only use clinical language, which is closer to practice but is also readily acceptable by students. Inculcating such values at an impressionable age is important. Any teaching in ethics can be undone in just a few moments by the physician's wrong bedside attitude.[11]

An international survey conducted by of medical ethics curricula in Asia by Mahaska, *et al*. (China, Hong Kong, Taiwan, Korea, Mongolia, Philippines, Thailand, Malaysia, Singapore, Indonesia, Sri Lanka, Australia, and New Zealand) showed a total of 89 medical schools offering some courses in which ethical topics were taught.[4] Separate medical ethics courses were mostly offered in all countries, and the structure of vertical integration was divided into four patterns. Most deans reported that physicians' obligations and patients' rights were the most important topics for their students. However, the evaluation was diverse for more concrete topics. It was concluded that offering formal medical ethics education is a widespread feature of medical curricula throughout the study area. A study of bioethics education in Japan revealed a lack of theoretical and organizational basis of interdisciplinary fields extending over medicine, humanities and the social sciences, a weakness that has been explained in terms of the gap between the structure of Japanese academic activity and the interdisciplinary character of ethics.[4,10] The point to ponder is whether there is any universal method of teaching ethics applicable worldwide to medical schools, especially those in non Western developing countries as the culture, religion, and practices are diverse and the content of the curriculum should be sensitive to this.

The paradigm shift from clinical examination-based to investigations-based treatment increases the importance of the role and responsibility of the paramedical staff. As the World Medical Association indicates in its latest document, some procedures, formerly performed by physicians, are now routinely done by medical technologists, nurses, and paramedics.[12] This results in physicians relying on data for diagnosis and the technician's role becoming increasingly important. In this changing scenario of increasing inter-connectedness between health care providers at different stages of the system, certain ethical principles need to be inculcated in students of medical, nursing, and paramedical courses so that the system holds together and remains trustworthy, while still providing a personal approach to patients as far as possible. At St.John's Medical College, based on a trial run of ethics courses given to paramedical students that included students of medical laboratory technology, perfusion technology, imaging technology, and renal dialysis, a syllabus was suggested for a total period of 24 hours and is being followed.[13]

Teaching ethics includes various methodologies ranging from the conventional lecture method to group discussions of case studies and narratives, problem-based learning, as well as the use of audio-visuals and films. The sessions should be designed to be as interactive as possible and maximum student participation must be encouraged. Similarly, films can be shown followed by a cine forum so that student reactions could be summed up to highlight a principle or compare differing viewpoints. In his assessment of the process of medical ethics education, Goldie found small group teaching to be highly acceptable to students and teachers.[1] Now there are many websites and discussion forums available for ethics discussion.

Finally, evaluation of performance in ethics is another important aspect to strengthen the role of ethics in medical education. Though the actual patient-doctor encounter can be simulated, it will be difficult to assess. Many medical students and physicians resent the unbalanced, highly theoretical approach taken in some traditional teaching programs in medical ethics.[14] Performance-based approaches are increasingly seen as crucial for the advancement of medical education, and this is no less true for teaching medical ethics. However, it will be a challenge to develop such evaluation modules. There is always a continuum between practice and education because a medical career is one of life-long learning. Medical ethics teaching and training should help the student at any level whatever may be the discipline to assimilate and conceptualize the basic principles of ethical reasoning.
